# Associations between preterm birth, small-for-gestational age, and neonatal morbidity and cognitive function among school-age children in Nepal

**DOI:** 10.1186/1471-2431-14-58

**Published:** 2014-02-27

**Authors:** Parul Christian, Laura E Murray-Kolb, James M Tielsch, Joanne Katz, Steven C LeClerq, Subarna K Khatry

**Affiliations:** 1 Department of International Health, Johns Hopkins Bloomberg School of Public Health, 615 N. Wolfe St, Room E2541, Baltimore, MD 21205, USA; 2 Department of Nutrition Sciences, The Pennsylvania State University, University Park, PA, USA; 3 The Nepal Nutrition Intervention Project-Sarlahi, Kathmandu, Nepal

**Keywords:** Low birth weight, Preterm, Birth asphyxia, Sepsis, Intelligence, Motor, Executive function

## Abstract

**Background:**

The long term consequences of low birth weight (LBW), preterm birth, small-for-gestational age (SGA, defined as birth weight for given gestational age less than the 10th percentile of the reference), and early newborn morbidity on functional outcomes are not well described in low income settings.

**Methods:**

In rural Nepal, we conducted neurocognitive assessment of children (n = 1927) at 7–9 y of age, for whom birth condition exposures were available. At follow-up they were tested on aspects of intellectual, executive, and motor function.

**Results:**

The prevalence of LBW (39.6%), preterm birth (21.2%), and SGA (55.4%) was high, whereas symptoms of birth asphyxia and sepsis were reported in 6.7% and 9.1% of children. In multivariable regression analyses, adjusted for confounders, LBW was strongly associated with scores on the Universal Nonverbal Intelligence Test (UNIT), tests of executive function, and the Movement Assessment Battery for Children (MABC). Preterm was not associated with any of the test scores. Conversely, SGA was significantly (all p < 0.005) associated with lower UNIT scores (−2.04 SE = 0.39); higher proportion failure on Stroop test (0.06, SE = 0.02); and lower scores on the backward digit span test (−0.16, SE = 0.04), MABC (0.98, SE = 0.25), and finger tapping test (−0.66, SE = 0.22) after adjusting for confounders. Head circumference at birth was strongly and significantly associated with all test scores. Neither birth asphyxia nor sepsis symptoms were significantly associated with scores on cognitive or motor tests.

**Conclusion:**

In this rural South Asian setting, intrauterine growth restriction is high and, may have a negative impact on long term cognitive, executive and motor function.

## Background

Neonatal conditions including intrauterine growth and gestational age at birth are strongly linked with neonatal and infant mortality. Worldwide there is a strong focus on reducing neonatal mortality through early life interventions to achieve the Millennium Development Goals in 2015 [1]. However, neonatal insults among survivors may also lead to adverse consequences in the long run that may pose a burden to families and societies. A majority of the neonatal insults may be of prenatal origin, especially those related to preterm birth and fetal growth restriction, the burden of which is the highest in low income countries. Newborns in developed countries who survive early life adverse events may receive appropriate interventions to overcome the long term neurodevelopmental impairments that may follow. However, in low income countries, these long term consequences may contribute substantially to the global burden of disability and human capital. Yet there is limited understanding of the long term consequences of neonatal insults among the surviving children. Investigations of sequelae and impairments have been done in a recent systematic review showing increased risk for learning difficulties, cognition or developmental delays, cerebral palsy, and hearing or visual impairment [2]. For example, preterm birth was associated with a 21% and sepsis with a 30% higher occurrence of cognitive or learning difficulties or development delay [2]. The majority of neonatal conditions examined in this analysis were severe, and mild-to-moderate symptoms were not considered. Additionally, their long term consequences were severe. Data were unadjusted for other environmental conditions and potentially confounding factors. In a recent study, two underlying biologic causes of low birth weight, preterm birth and intrauterine growth restriction were evaluated for their effects on cognitive outcomes among Swedish children at 5–8 y of age [3]. Using the Wechsler Scale for Intelligence this study revealed a higher risk of cognitive impairment among children who were very preterm and intrauterine growth restricted but not among those very preterm alone [3]. The mechanisms of the effects of these intrauterine insults have been postulated and include abnormal fetoplacental blood flow [4,5] and differences in brain size and morphology [6]. Brain sparing, generally observed with intrauterine growth restriction, and assessed using decreased pulsatility index in the middle cerebral artery in the fetus has previously been shown to be associated with impaired cognitive function in preschool years [7].

In rural Nepal in 2007–2009 we conducted a longitudinal follow-up of children to examine prenatal and postnatal supplementation with micronutrients on school age neurocognitive function [8-10]. In this cohort of children we have prospectively collected data on birth size, gestational duration, and neonatal morbidity. In the present analysis we examine the long term consequences of low birth weight, preterm birth, small-for-gestational age, and neonatal sepsis and birth asphyxia on cognitive and motor function in surviving school age children 7–9 y of age.

## Methods

The population-based studies described below were all conducted as part of the Nepal Nutrition Intervention Project - Sarlahi (NNIPS) in rural southern plains (sea level) of Nepal where 90% of births occurred at home and only 2-3% assisted by skilled birth attendants. Children born to women who participated in a double-blind randomized controlled trial (RCT) of micronutrient supplementation during pregnancy (1999–2001) [11] and who themselves were participants in a RCT of micronutrient supplementation during their preschool years (2001–2005) [12] were tracked and followed at 7–9 y of age (2007–2009) to conduct assessments of general intelligence, and executive and motor function. In both studies randomization was done by geographically defined clusters (sectors, n = 426).

Details of the follow-up assessments and long term effects of prenatal and postnatal micronutrient supplementation have been published previously [8,9]. By design we chose only selected arms of the maternal and child supplementation trials, specifically to examine the role of iron-folic acid and zinc in motor and cognitive development. Briefly out of 3675 who had participated in both trials, 1748 belonged to non-selected study arms and were excluded, whereas 1927 children in the purposively selected study arms were visited in their homes and invited to participate in the prospective follow-up study, following parental verbal consent and child assent. Two home-based visits provided information about demographics, socioeconomic status (SES), child’s school enrolment history, morbidity over the previous 7- and 30-days, dietary intake, maternal literacy, and household salt iodine content. As part of the SES questions, data on household ownership of eleven assets was collected. Assets included both materials and domestic animal ownership.

A battery of psychometric tests was administered in a central clinic by trained and certified psychological testers. General intelligence was assessed with the Universal Nonverbal Intelligence Test (UNIT) [13]. A (numbers) Stroop test [14], and backward digit span (Wechsler and Psychological Corporation. 1991) were utilized to assess inhibitory control and verbal working memory, components of executive functioning. The Movement Assessment Battery for Children (MABC) and a finger tapping test were administered to evaluate gross and fine motor abilities [15]. Stimulation at home was measured with the Middle Childhood Home Observation for the Measurement of the Environment (HOME) and the mother’s reasoning abilities with the Raven’s Colored Matrices [16,17]. Anthropometry (weight, height and mid upper arm circumference) and hemoglobin (using the HemoCue machine) were also assessed at the clinic.

### Statistical analysis

The present analysis was done on all children followed-up for the assessment across the original maternal and child supplementation groups. Data on birth measurements and morbidity assessed in the original maternal intervention trial were merged with the data collected at the time of follow-up. Anthropometric measurements were done in the home where a majority of the births occurred. Because weight was measured at variable times since birth and since about 7% of infants were missing weight measurements, 34% of whom died very soon after birth, we developed a measurement error model that allowed us to estimate the weight of the baby at birth for measurements made at variable times after birth and to impute missing measurements [18,19]. Missing birth weights were imputed using a multiple imputation method [20]. Women of child-bearing age were visited monthly and asked to recall the date of last menstrual period. If they had not menstruated in the past 5 weeks, a urine based pregnancy test was administered. Gestational age of the newborn was assessed using a combination of date of last menstrual period and date of positive pregnancy test [11]. Data on newborn complications and morbidity were assessed soon after birth and daily for the first 10 days of life to elicit a 24-h maternal history of 9 infant morbidity symptoms [21].

Exposure variables were defined as low birth weight < 2500 g, preterm and very preterm birth as gestational age < 37, and < 34 completed weeks, small for gestational age (SGA) defined as birth weight less than the 10th percentile for a given gestational age and sex using the Alexander US-fetal growth reference [22]. Sepsis was defined as at least 2 consecutive days of 2 of the following symptoms: 1) inability or difficulty suckling, 2) lethargy, 3) difficulty breathing, 4) convulsions or stiffness of back, 5) hot to touch or temp > 100°F, 6) cold to touch or temp < 96.6°F, 7) red purulent umbilicus [21]. Birth asphyxia was defined as inability to cry at birth and one of the following symptoms: 1) inability to breathe at birth, 2) convulsions or spasms, or 3) inability to suckle normally after birth [23]. Data were analyzed with all maternal and child intervention groups combined for two reasons - one, combinations of micronutrients in the maternal supplementation trial was shown to impact some of the exposure variables in this analysis and was deemed as being in the causal pathway [11,21] and two, child supplementation with iron-folic acid and/or zinc had no impact on the outcomes [9].

Bivariate analyses were done to examine the relationship between exposures and outcomes across each of the psychometric tests. Similarly, household, environmental and maternal factors were examined for their association with the outcomes to identify potential confounders. A simple socioeconomic index was created by summing up household asset ownership which ranged from 0–11. Child age, sex, asset score, HOME score, child schooling, previous 7-d intake of different food groups, and history of diarrhea/dysentery, lower respiratory infection, maternal literacy, Raven's score, and household salt iodine ≥15 ppm were examined as potential confounders due to their association with the outcomes. The raw UNIT test scores were converted to *T* scores, and the Stroop test was expressed as a proportion of failures (i.e. those who failed the practice test). The MABC test is scored based on failures and thus higher scores reflect worse instead of better performance. Raw scores on each task were converted to scaled scores. For the backward digit span, the longest number of digits remembered correctly was used.

Multivariable linear regression models were run to adjust for confounders using a backward stepwise selection procedure with a significance level for removal from the model of 0.1. All exposure variables were locked into the models. Once the significant variables were identified we then applied the generalized estimating equations approach to the multivariable linear regression models to adjust for geographic clustering. The final models only retained those confounders that were significant at p < 0.05. Stata version 11.0 (StataCorp, College Station, TX) and SAS version 9.2 (SAS Institute, Cary, NC) were used to analyze data.

Ethical approval for the study was provided by the institutional review boards at the Institute of Medicine, Tribhuvan University in Nepal and Johns Hopkins School of Public Health and The Pennsylvania State University in the US.

## Results

Out of 1927 children eligible for the study, 93-94% were successfully assessed. Losses to follow-up were due to refusals (n = 14), families having moved out of study area or not met upon repeated visits (n = 75), and child death (n = 3). The prevalence of low birth weight, preterm birth and SGA in this sample of children was 39.6%, 21.2%, and 55.4%, respectively (Table 1). Half (50.2%) of the children were male, and 80% had ever been sent to school. Maternal literacy (ability to read or write) in this setting was 21.7% and child diet as reflected by 7-day food frequency data indicated children had consumed at least once of a variety of foods in the past week. The mean height for age Z score was −1.88 (SD = 0.87), and BMI Z score was −1.18 (SD = 0.82). The mean age of children at follow-up was 8.4 years (SD = 0.66). Mean (SD) scores on cognitive and motors tests by exposure categories are shown in Table 2. Mean test scores on cognitive, executive and motor function tests were generally worse in the preterm, SGA, low birth weight, and sepsis groups but not the birth asphyxia group. The unadjusted coefficients were significant for preterm birth, SGA, LBW, birth asphyxia (albeit in the opposite direction) and head circumference for the UNIT and the tests of executive function (Table 3). In the adjusted analyses, preterm birth was not significantly associated with these tests. After adjusting for child age, diet, diarrheal and acute lower respiratory morbidity, schooling, maternal literacy, parity, Raven's score, and household socioeconomic variables, SGA was associated with a lower score (−2.04) on the test of intelligence, a higher failure (6%) on the Numbers Stroop test, and a mean 0.2 score lower backward digit span. A one cm increase in head circumference at birth was associated with an adjusted mean *T* score on the UNIT of 0.44 and an improved performance on the tests of executive function (all p < 0.05). Preterm birth, sepsis and birth asphyxia were not significantly associated with any of the test scores. In the adjusted analysis SGA and LBW were associated with a higher (worse) score on the MABC of 0.98 and l.29 (p < 0.001) (Table 4). Birth asphyxia, but not sepsis, was associated with a 1.46 higher (worse) score on the MABC test (p = 0.04). Every one cm increase in head circumference was associated with a lower (better) MABC score (p < 0.001) in the adjusted analysis. For the finger tapping test, only SGA was significantly associated with a lower score (p = 0.004) in the adjusted analysis. Very preterm birth was examined but was not associated with the outcomes (data not shown).

**Table 1:** Conditions at birth and characteristics of children at 7–9 y of age

	**N**	**n**	**%**
**Conditions at birth**			
Preterm (<37 wk)	1923	408	21.2
Very preterm (< 34 wk)	1923	124	6.4
Small for gestational age^1^	1900	1052	55.4
Low birth weight (<2500 g)	1927	764	39.6
Sepsis^2^	1827	167	9.1
Birth asphyxia^3^	1823	123	6.7
**Characteristics at follow-up**			
Male	1927	968	50.2
Ever been to school	1835	1467	80.0
Maternal literacy	1912	415	21.7
Child morbidity and diet (any intake in past 7 d)			
Lower respiratory infection^4^	1830	41	2.2
Diarrhea^5^	1835	68	3.7
Diet in the past 7 d			
Milk and dairy^6^	1834	1309	71.4
Meat, chicken, fish	1834	1080	58.9
Dark green leafy vegetables	1834	1279	69.7
Citrus fruits^7^	1833	774	42.2
Yellow fruits and vegetables^8^	1834	720	39.3
	N	Mean	SD
Child age, y	1927	8.4	0.66
Child HAZ	1822	−1.88	0.87
Child BMIZ	1822	−1.18	0.82
Child Hemoglobin (g/dl)	1823	12.8	5.5
HOME score^9^	1821	24.0	6.2
Asset score^10^	1830	4.5	2.2
Maternal score on Raven's test	1752	16.6	5.2

^1^Small for gestational age defined as birth weight less than the 10th percentile for a given gestational age and sex using the Alexander US-fetal growth reference (22).

^2^Sepsis defined as at least 2 consecutive days of 2 of the following symptoms: 1) inability or difficulty suckling, 2) lethargy, 3) difficulty breathing, 4) convulsions or stiffness of back, 5) hot to touch or temp > 100 F, 6) cold to touch or temp < 96.6 F, 7) red purulent umbilicus.

^3^Birth asphyxia defined as inability to cry at birth and one of the following symptoms: 1) inability to breathe at birth, 2) convulsions or spasms, or 3) inability to suckle normally after birth.

^4^Productive cough or rapid breathing and fever.

^5^Watery stools ≥4 times/d or blood in stool.

^6^Includes milk, yogurt and buttermilk.

^7^Includes oranges and guava.

^8^Includes ripe mango, papaya, jackfruit, pumpkin.

^9^Asset score ranges from 0 to 11 and is made up of any ownership of goats, cattle, cart, bicycle, motorcycle, electricity, radio, TV, telephone, mobile phone, watches in the household.

^10^Using the HOME inventory.

*HAZ* Height for age Z score.

*BMIZ* Body mass index Z score.

**Table 2:** Mean (SD) scores on cognitive and motor tests in children 7–9 y of age by conditions at birth

	**UNIT**		**Numbers stroop, failure**		**Backwards digit**		**MABC score**		**Finger tapping test**	
	**(n = 1793)**		**(n = 1812)**		**(n = 1814)**		**(n = 1782)**		**(n = 1810)**	
	**Mean**	**SD**	**Mean**	**SD**	**Mean**	**SD**	**Mean**	**SD**	**Mean**	**SD**
Not preterm	50.3	10.0	0.30	0.46	1.94	1.12	8.43	6.14	36.6	5.3
Preterm	48.6	9.8	0.37	0.48	1.71	0.99	9.04	6.30	36.3	5.1
Not SGA	51.3	10.3	0.28	0.45	2.00	1.14	7.85	5.82	37.0	5.3
SGA	49.0	9.7	0.35	0.48	1.81	1.06	9.16	6.40	36.0	5.3
Not LBW	51.6	10.1	0.25	0.43	2.05	1.16	7.62	5.59	36.9	5.1
LBW	47.6	9.4	0.42	0.49	1.66	0.95	9.98	6.73	35.8	5.4
No sepsis	50.1	10.1	0.32	0.46	1.91	1.11	8.53	6.14	36.6	5.2
Sepsis	49.7	9.6	0.38	0.49	1.75	0.98	8.76	6.61	35.9	5.7
No birth asphyxia	49.9	10.0	0.32	0.47	1.89	1.1	8.46	6.06	36.5	5.2
Birth asphyxia	52.2	10.6	0.31	0.46	1.97	1.14	9.62	7.50	36.7	5.9

UNIT: Universal Nonverbal Test of Intelligence.

MABC: Movement Assessment Battery for Children.

Preterm defined as gestational age <37 weeks.

Low birth weight defined as <2500 g.

Small for gestational age (SGA) defined as birth weight less than the 10th percentile for a given gestational age and sex using the Alexander US-fetal growth reference (22).

Sepsis defined as at least 2 consecutive days of 2 of the following symptoms: 1) inability or difficulty suckling, 2) lethargy, 3) difficulty breathing, 4) convulsions or stiffness of back, 5) hot to touch or temp > 100 F, 6) cold to touch or temp < 96.6 F, 7) red purulent umbilicus.

Birth asphyxia defined as inability to cry at birth and one of the following symptoms: 1) inability to breathe at birth, 2) convulsions or spasms, or 3) inability to suckle normally after birth.

**Table 3:** Unadjusted and adjusted differences in test scores for general intellectual and executive function in children 7–9 y of age by conditions at birth

	**UNIT**				**Numbers stroop, failure**				**Backward digit span**			
	**Unadj diff (SE)**	**p-value**	**Adj diff (SE)**	**p-value**	**Unadj diff (SE)**	**p-value**	**Adj diff (SE)**	**p-value**	**Unadj diff (SE)**	**p-value**	**Adj diff (SE)**	**p-value**
Preterm	−1.70 (0.58)	0.003	−0.07 (0.46)	0.89	0.06 (0.03)	0.02	−0.00 (0.02)	0.82	−0.23 (0.06)	<0.001	−0.06 (0.05)	0.22
SGA	−2.37 (0.48)	<0.001	−2.04 (0.39)	<0.001	0.06 (0.02)	0.01	0.06 (0.02)	<0.001	−0.19 (0.05)	0.008	−0.16 (0.04)	<0.001
LBW	−4.00 (0.47)	<0.001	−1.63 (0.40)	<0.001	0.17(0.02)	<0.001	0.06 (0.02)	<0.001	−0.38 (0.05)	<0.001	−0.16 (0.04)	<0.001
Sepsis	−0.34 (0.84)	0.69	0.22 (0.60)	0.71	0.07 (0.04)	0.08	0.04 (0.03)	0.19	−0.17 (0.09)	−0.07	−0.04 (0.07)	0.62
Birth asphyxia	2.26 (1.00)	0.02	1.32 (0.81)	0.10	−0.01 (0.05)	0.77	0.03 (0.04)	0.36	0.08 (0.11)	0.45	−0.00 (0.09)	0.97
Head circumference, cm	1.11 (0.14)	<0.001	0.44 (0.14)	<0.001	−0.04 (0.01)	<0.001	−0.01 (0.01)	0.02	0.10 (0.02)	<0.001	0.04 (0.01)	0.007

Adjusted for significant confounders in each model from the following ones using backward stepwise multivariable linear regression analysis and GEE for adjustment for clustering : child age, sex, ever started school, intake of dark green leafy vegetables, intake of citrus, maternal literacy, maternal Raven’s score, parity, asset score, history of diarrhea/dysentery, household salt iodine level.

**Table 4:** Unadjusted and adjusted differences in test scores for motor function in children 7–9 y of age by conditions at birth

	**MABC**				**Finger tapping test**			
	**Unadj diff (SE)**	**p-value**	**Adj diff (SE)**	**p-value**	**Unadj diff (SE)**	**p-value**	**Adj diff (SE)**	**p-value**
Preterm	0.61 (0.36)	0.09	0.11 (0.31)	0.72	0.26 (0.30)	0.40	0.02 (0.26)	0.95
SGA	0.94 (0.32)	0.003	0.98 (0.25)	<0.001	0.82 (0.27)	0.003	−0.66 (0.22)	0.004
LBW	2.36 (0.29)	<0.001	1.29 (0.31)	<0.001	1.1 (0.25)	<0.001	−0.25 (0.23)	0.26
Sepsis	0.23 (0.52)	0.66	−0.26 (0.48)	0.60	0.67 (0.44)	0.12	−0.00 (0.40)	1.00
Birth asphyxia	1.16 (0.61)	0.06	1.46 (0.70)	0.04	−0.23 (0.52)	0.66	−0.10 (0.51)	0.85
Head circumference, cm	0.57 (0.08)	<0.001	−0.33 (0.08)	<0.001	0.10 (0.07)	0.003	0.02 (0.07)	0.81

Adjusted for significant confounders in each model from the following ones using backward stepwise multivariable linear regression analysis and GEE for adjustment for clustering: child age, sex, ever started school, intake of dark green leafy vegetables, intake of citrus, maternal literacy, maternal Raven’s score, parity, asset score, history of diarrhea/dysentery, household salt iodine level.

## Discussion

In this rural, Nepalese, community-based prospective study of neurocognitive assessment in children 7–9 y of age, we were able to demonstrate the long term adverse consequences of intrauterine insults and neonatal morbidity. In this setting the prevalence of low birth weight was high at 39% with a high burden of intrauterine growth restriction, one of the contributors of LBW as reflected in SGA (55%). We found that SGA was strongly associated with negative consequences for outcomes of general intelligence and executive and motor function in this environment. Preterm birth which also contributes to LBW was not associated with cognitive and functional impairments. Neither symptoms of sepsis nor birth asphyxia were linked to the outcomes except in the case of motor scores, which were modestly although significantly worse among children who had experienced symptoms of birth asphyxia.

Preterm birth is the second largest direct cause of under 5 child mortality and it is estimated that 14.9 million babies were born preterm worldwide in 2010 [24]. In a recent systematic review preterm birth has been shown to be associated with a 21% higher risk of cognitive or learning difficulties or developmental delay [2]. In our study we did not see this elevated risk in the adjusted analyses. It may be that the systematic review did not adequately control for confounding factors or that the studies were largely derived from high income settings where survival in very preterm and preterm infants is high related to advanced neonatal care, unlike in a setting such as Nepal where 90% of newborns were born at home. A recent meta-analysis which included the present Nepali cohort among others has shown that the risk of infant mortality related to preterm birth is high in low and middle income countries and higher than related to SGA (25) suggesting that the most vulnerable children do not survive the deleterious effects of being born prematurely. Our examination of the neurocognitive outcomes in those born very preterm (<34 weeks) found no association both perhaps due to the lower sample size in this exposure category and an even higher mortality experience among these children. SGA, on the other hand, while occurring at a much higher prevalence was not be associated with as high a mortality risk as preterm birth in low and middle income settings [25]. We this cohort we have previously shown that over twice as many children are likely to die if they are born preterm alone compared to those born SGA alone [26]. However, future studies are needed to better understand long term consequences of preterm birth on school achievement and IQ in these environments.

Hypoxic ischemic encephalopathy and sepsis have previously been each associated with a 30% higher cognitive or learning difficulties or developmental delay [2]. In our study, the association was absent or very modest. This may be explained by limitations with our exposure data. The first major limitation was that in our study setting in which births occurred in the home, our assessment of neonatal morbidity and complications were based on maternal reports in the first 10 days of life. Although our data collectors were highly trained and used standardized questionnaires to elicit morbidity histories every 24 hours, our data may suffer from reporting bias and misclassification. Yet, we used previously validated algorithms for defining sepsis and birth asphyxia [21,23,27]. The other consideration is that hospital-based, physician diagnosed conditions may be very likely to identify severe morbidity, and emergency interventions may have resulted in improved survival, whereas our self-reported morbidity symptoms among surviving neonates were likely less severe.

Small-for-gestational age at birth is not a perfect measure of intrauterine growth restriction (IUGR). However, our findings are similar to a recent study in which true IUGR was assessed using abnormal umbilical artery blood flow using Doppler velocimetry [3]. This study found only very preterm with IUGR but not preterm alone to have an increased risk of cognitive impairment at early school age [3]. In addition, our study found a strong association between head circumference, a measure of brain development that is also strongly associated with fetal growth and school age cognitive performance, after adjustment for multiple concurrent home, environment and biologic factors. Unlike preterm which has numerous life style, infection and environmental causes, SGA has long been known to be strongly caused by maternal nutritional factors, specifically maternal pre-pregnancy height, BMI, and weight gain during pregnancy [28] all of which are linked to both short and long term macro and micronutrient status of women of reproductive age. In Nepal, maternal undernutrition and micronutrient deficiencies during pregnancy are highly prevalent [11,29]. In the same Nepali cohort of children, we previously showed that maternal supplementation with iron-folic acid during pregnancy had a significant positive effect on aspects of general intelligence, and executive and motor function [8], demonstrating the importance of fetal requirements of iron for CNS growth and development.

Strengths of the study include a large sample size, assessment of a range of tests of functional outcomes, and high follow-up rates. We also adjusted for a large number of potential confounders and factors that influence childhood cognitive function. Limitations include lack of data on postnatal growth, morbidity and diet including breastfeeding practices. Additionally, the analysis suffered from survival bias.

## Conclusion

Our analysis reveal the adverse consequences of poor fetal growth on outcomes of cognitive, motor and executive function in a low income setting in South Asia. Our findings reveal that the central nervous system and its development may be susceptible to fetal life nutritional deprivation. Although intrauterine insults leading to poor fetal growth may result in only modest increases in the risk of immediate mortality, the long term consequences on cognitive function may have substantial public health implications for the health of future generations and should be assessed in future studies in low and middle income settings. Nutritional interventions for women of reproductive age before and during pregnancy as well as psychosocial interventions targeted to low birth weight or small-for-gestational age infants may be important public health strategies for ameliorating the long term adverse consequences of intrauterine growth restriction in under resourced countries.

## Abbreviations

BMI: Body mass index; CNS: Central nervous system; IUGR: Intrauterine growth restriction; LBW: Low birth weight; MABC: Movement Assessment Battery for Children; SGA: Small-for-gestational age; UNIT: Universal Non-verbal intelligence test.

## Competing interests

The authors declare that have no competing interests.

## Authors’ contributions

PC was the principal investigator and was involved with the study concept and design, and analysis and writing and editing the manuscript. LEMK was involved with study concept and design, acquisition of data, analysis and interpretation of data, obtaining funding, administrative/technical/material support and edited the manuscript. JMT was involved with study concept and design, interpretation of data, and revision of manuscript. JK was involved with study concept and design, statistical analysis and revision of manuscript. SLC was involved in review of manuscript, administrative/technical/material support, and study supervision. SKK was involved with administrative/technical/material support, acquisition of data, and review of manuscript. All authors read and approved the final manuscript.

## Pre-publication history

The pre-publication history for this paper can be accessed here:

http://www.biomedcentral.com/1471-2431/14/58/prepub
